# What does patient engagement mean for Canadian National Transplant Research Program Researchers?

**DOI:** 10.1186/s40900-018-0096-0

**Published:** 2018-04-09

**Authors:** Julie Allard, Fabián Ballesteros, Samantha J. Anthony, Vincent Dumez, David Hartell, Greg Knoll, Linda Wright, Marie-Chantal Fortin

**Affiliations:** 1 Centre de recherche du Centre hospitalier de l’Université de Montréal (CRCHUM), 900 Saint-Denis St., Room 12-454, Montréal, QC, H2X 0A9 Canada; 2 Canadian National Transplant Research Program, Edmonton, Canada; 3 Child Health Evaluative Sciences, The Hospital for Sick Children, Toronto, Canada; 4 Factor-Inwentash Faculty of Social Work, University of Toronto, Toronto, Canada; 5 Direction collaboration et partenariat patient, Faculty of Medicine, Université de Montréal, Montréal, Canada; 6 Centre of Excellence on Partnership with Patients and the Public, Université de Montréal, Montréal, Canada; 7 Ottawa Hospital Research Institute, Ottawa, Canada; 8 Department of Surgery and Joint Centre for Bioethics, University of Toronto, Toronto, Canada; 9 Université de Montréal, Montréal, Canada

**Keywords:** Patient engagement in research, Organ transplantation, Organ donation, Researchers’ perspectives, Qualitative methodology

## Abstract

**Plain English summary:**

In recent years, the importance of involving patients in research has been increasingly recognized because it increases the relevance and quality of research, facilitates recruitment, enhances public trust and allows for more effective dissemination of results. The Canadian National Transplant Research Program (CNTRP) is an interdisciplinary research team looking at a variety of issues related to organ and tissue donation and transplantation. The aim of this study was to gather the perspectives of CNTRP researchers on engaging patients in research.

We conducted interviews with 10 researchers who attended a national workshop on priority-setting in organ donation and transplant research. The researchers viewed patient engagement in research as necessary and important. They also considered that patients could be engaged at every step of the research process. Participants in this study identified scientific language, time, money, power imbalance, patient selection and risk of tokenism as potential barriers to patient engagement in research. Training, adequate resources and support from the institution were identified as facilitators of patient engagement.

This study showed a positive attitude among researchers in the field of organ donation and transplantation. Further studies are needed to study the implementation and impact of patient engagement in research within the CNTRP.

**Abstract:**

**Background**

Involving patients in research has been acknowledged as a way to enhance the quality, relevance and transparency of medical research. No previous studies have looked at researchers’ perspectives on patient engagement (PE) in organ donation and transplant research in Canada.

**Objective**

The aim of this study was to gather the perspectives of Canadian National Transplant Research Program (CNTRP) researchers on PE in research.

**Methods**

We conducted semi-structured interviews with ten researchers who attended a national workshop on priority-setting in organ donation and transplant research. The interviews were digitally recorded and transcribed verbatim, and the transcripts were subjected to qualitative thematic and content analyses.

**Results**

The researchers viewed PE in research as necessary and important. PE was a method to incorporate the voice of the patient. They also considered that patients could be engaged at every step of the research process. The following were identified as the main barriers to PE in research: (i) scientific jargon; (ii) resources (time and money); (iii) tokenism; (iv) power imbalance; and (v) patient selection. Facilitating factors included (i) training for patients and researchers, (ii) adequate resources and (iii) institutional support.

**Conclusion**

This study revealed a favourable attitude and willingness among CNTRP researchers to engage and partner with patients in research. Further studies are needed to assess the implementation of PE strategy within the CNTRP and its impact.

## Introduction

In recent years, the patient’s involvement in research has extended beyond the role of research participant. In order to take into account the experiential knowledge of patients, there is a tendency to actively engage patients in the research process. As described by the Canadian Institutes of Health Research in their Strategy for Patient-Oriented Research, “patient engagement occurs when patients meaningfully and actively collaborate in the governance, priority-setting, and conduct of research, as well as in summarizing, distributing, sharing, and applying its resulting knowledge (i.e., the process referred to as ‘knowledge translation’).” [1] “Patient” is “an overarching term inclusive of individuals with personal experience of a health issue and informal caregivers, including family and friends.” [2] Patient engagement (PE) in research increases the relevance and quality of research, facilitates recruitment of participants and knowledge transfer, and enhances public trust, transparency and accountability. [3–8] PE can take the form of consultation, collaboration and participant-led research. [9]

The Canadian National Transplant Research Program (CNTRP) is a national research initiative designed to increase organ and tissue donation in Canada, improve graft survival and enhance the quality of life of Canadians living with a transplant. [10, 11] The program brings together more than 150 funded and active researchers, patients and trainees in the field of donation and transplantation of solid organs and hematopoietic cells. The CNTRP aims to increase PE in research and support novel approaches that integrate patients and families as active participants across the research network. To achieve this goal of PE, the CNTRP conducted a pilot workshop in 2014 in French with 10 patients and 5 researchers, in Montréal, Quebec, to identify research priorities. [12] To refine the preliminary research priorities identified during the workshop, elicit new priorities from a broader national cohort of patients, and rank the priorities in order of importance, a national survey and a larger, national workshop with patients, caregivers, health care professionals and researchers were conducted in 2015.

Researchers’ attitudes toward PE in research could influence the strategy for engaging patients. It has been demonstrated that researchers having a positive attitude toward PE is a key component of a successful partnership, whereas researchers having a negative attitude could be associated with tokenism. [13] To develop and implement a strategy for PE in research within the CNTRP, it is important to understand researchers’ attitudes. This exploratory and qualitative study aimed to describe CNTRP researchers’ perspectives on PE before and after their participation in a national priority-setting workshop with patients, caregivers and clinicians.

## Methods

We used the consolidated criteria for reporting qualitative studies checklist. [14] This study was exploratory in nature and used semi-structured interviews with CNTRP researchers. All 11 researchers who agreed to participate in the CNTRP’s national priority-setting workshop were invited to participate by email. These researchers were all involved in organ and stem cell donation or transplantation, with various areas of scientific inquiry and research expertise (clinical, biomedical, health organization, etc.). Ten researchers agreed to participate in pre- and post-workshop interviews. The pre-workshop interviews were conducted 1 to 14 days before the workshop (between November 11 and 24, 2015). The post-workshop interviews were carried out 23 to 72 days after the workshop (between January 19 and March 8, 2016). Nine of the researchers’ pre and post interviews were conducted by phone, and one’s pre and post interviews were conducted in person. The number of participants allowed data saturation for pre- and post-workshop interviews to be achieved (no new code was created after the eighth interview). [15] A member of the research team (FB) who has experience conducting qualitative interviews and was present at the national workshop conducted all of the interviews. The interviews lasted approximately 20 minutes and were digitally recorded. The Centre hospitalier de l’Université de Montréal research ethics board approved the study and all participants provided informed consent.

The topics covered during the interviews were outlined in an interview guide with open-ended questions. The pre-workshop interview questions addressed the following themes: (i) researchers’ perspectives on PE in research; (ii) previous experience with PE in research; (iii) appropriate methods to engage patients in research; (iv) anticipated impact and benefits of PE for research; (v) barriers to PE in research; and (vi) socio-demographic characteristics. During the post-workshop interviews, the following themes were addressed with the researchers: (i) their experience during the workshop; (ii) the impact of the workshop on their perspectives on PE in research; (iii) their intention to engage patients in their own research; and (iv) anticipated facilitators of and barriers to PE in research. The two interview guides were pre-tested with three individuals. Consistent with qualitative methodology, the interview guide was modified throughout the study as new topics emerged from the interviews.

The interview transcripts were analyzed using the content and thematic analysis method described by Miles and Huberman. [16] This involved: (i) establishing a list of themes based on the interview guide, which constituted the coding frame; (ii) reading the transcripts and sorting them according to the coding frame to create a more abstract frame of analysis; (iii) adding new themes or categories as they emerged from the transcripts; (iv) organizing these categories into figures, charts or matrices; and (v) drawing corresponding conclusions. NVivo 11 (QSR International) computer software was used to facilitate the qualitative analysis. An independent researcher with experience in qualitative methods and research in the field of organ transplantation (JA) coded 15% of the raw data, and the rate of coding agreement was subsequently assessed at 97%.

## Results

### Participant characteristics

Ten participants from four Canadian provinces agreed to participate. Researchers came from different research fields: kidney transplantation, basic sciences, multi-organ transplantation, organ donation, and legal and ethical issues. The characteristics of the participants are summarized in Table 1.

**Table 1:** Participant characteristics

Characteristics	*N* = 10
Gender	
Male/Female	6/4
Research field	
Kidney transplantation	4
Basic sciences	3
Multi-organ transplantation	1
Donation (brain death and end of life)	1
Legal and ethical issues	1
Province	
Ontario	4
Alberta	3
British Columbia	2
Quebec	1
Previous experience with PE	4

### Researchers’ perspectives on PE before the workshop with patients

At the beginning of the interview, researchers were asked about any previous experience with PE in research. Four participants reported past experiences of PE in research that were “*rewarding*” because it offered new insight and reminded them that patient outcomes should be at the centre of medical research. Their experiences ranged from asking patients for their opinions or having them participate in forums and group discussions, to engaging them in the early stages of a research project (e.g. research design). One researcher reported patients’ eagerness to be involved in the research process, but also their concerns with the commitment required; *“[E]nthusiasm was incredibly high, and there were two things that happened with the patients: one was that the patients felt very honoured and excited that their input was being solicited; the other […] was that we actually included them on a number of phone calls and I think they felt quite lost sometimes and not useful, and overwhelmed.”* (R06).

All of the researchers interviewed had a positive attitude toward PE in research, feeling that this approach is *“necessary and crucial for research”* (R01) because patients need to have a voice in what is given priority in research, just as they have a say in their personal health care. In a context where resources and funds are scarce, research needs to focus on topics relevant to patients. “*We should be using [the] scarce research dollars to study and understand questions that are relevant to our patients.”* (R03) Researchers felt that patients’ needs and research priorities could differ from their own, mentioning that *“researchers and patients have fairly different priorities.”* (R05) They also indicated a preference for collaborative relationships in which patients are actively involved and engaged in the research process, rather than consultative relationships.

#### Benefits and anticipated impact of PE in research

PE in research was viewed as important given its benefits for the patients, the researchers and the research itself.

For some participants, PE in research is beneficial to patients as it can empower them by acknowledging their expertise and experiential knowledge. By having the opportunity to express their opinions, they could influence the setting of research priorities, as stated by one of the researchers: *“As far as the research priorities, I think it gives the patient an opportunity to express to the transplant community in this case what’s important to them, and what would make a huge difference in their lives.”* (R04) PE in research could also allow patients to gain a better understanding of what medical research is all about, become part of the research team and participate in the decision-making process in research. As an example, one respondent explained that *“it’s a rewarding experience, and so that’s how […] involving patients, having them understand what the research questions are, and then kind of commenting on the relevance of those, and adding their own perspective is both important and useful but also rewarding.”* (R05).

Researchers noted that PE in research is also beneficial for them because it gives meaning to what they are doing and connects their research to the public thus *“humanizing research.”* Researchers felt that PE in research can help them feel comfortable with the direction of their research program and identify limitations in their projects. Patients could validate research themes and approaches, as well as provide new ones. These benefits are clearly identified in the words of the researchers who had experience with PE in research: *“It is very rewarding for both the patient and […] for the research team. New perspectives were brought up, or the researchers got confirmation that their questions are relevant and important and then viewed positively by the patient.”* (R05).

Lastly, PE is also beneficial for research because it could improve researchers’ skill level in communicating with the public and future research participants, in addition to helping patients become better advocates for research. According to one respondent*, “the lay public also becomes [a] tremendous [advocate] both in the media and among the larger community for research, because they become invested in it in a way that the medical health professionals and professional researchers [cannot, being] a little bit more divorced from directly accessing that community other than as guests in the community.”* (R03).

#### Types of research and patients’ roles

During the interview, researchers were asked about what kind of research was most appropriate for engaging patients and in which aspects of research patients may have a role to play. They were divided on which types of research patients should be involved in. Half of the participants said patients could engage in any type of research *(“I see very few studies where it wouldn’t be helpful”* [R01]), while the other half believed it would be difficult or inappropriate to engage them in basic sciences research, as exemplified in this excerpt*: “[P]atient engagement isn’t going to apply in every setting and situation. […] I could see how a basic scientist is going to see very little use for this. So again it depends on the perspective and the type of research.”* (R06) The latter type of research is seen as posing major challenges due to design and procedural complexity. However, some researchers thought that patients and researchers could learn from a dialogue and foresee the outcome of such research. *“I think it would be good to have some dialogue with the patients to teach them about what we’re doing in the fundamental science, to see where we’re heading, and learning from them what they think would be important where we’re heading; it’s easier to kind of make adjustments early on in the research, rather than we finally get to the clinical part and they say, well this is not really what we’re looking for at the moment.”* (R10) Clinical research is seen as a first choice to start engaging patients*, “I think probably more so in medical or clinical research as opposed to basic science.”* (R08).

When asked about what aspects of research they think patients have a role to play in, 9 out of 10 researchers thought patients could be involved in every step of the research process. That being said, they all agreed about engaging patient in priority-setting exercises and study design. They also envisioned a role for PE in knowledge transfer and dissemination. One researcher even suggested engaging patients in writing a section of a grant proposal. Others mentioned having participants involved in recruiting research participants at stages of the research process, to engage with patients. *“[Patients] have a role to play in designing the research program to some extent, not necessarily the scientific aspect of it but certainly ensuring that the research is being done in a respectful way, and that the procedures are feasible for potential participants in the research.”* (R03).

#### Anticipated barriers

Before the national workshop, researchers were asked about the barriers to PE in research. The main barriers identified by researchers were resources and workload, language, risk of tokenism, patient selection, and power imbalance. Table 2 summarizes the barriers with discussion excerpts.

**Table 2:** Anticipated barriers to PE in research pre- and post-workshop

Theme	Pre-workshop	Post-workshop
Resources and workload	*“I think it’s a lot of work to bring patients up to speed on the process and the content; it takes a lot of work. And it’s unclear whether the benefits of putting that much time and effort in are going to add value.”* (R01) *“I think it also creates some encumbrances that need to be balanced, […]. I think it does also create some challenges for the researchers to fully develop a project in a timely way and get it funded at the level that they’d like to get it funded,* etc.*”* (R03)	*“I think many of these patients have full-time jobs and therefore trying to engage them during working hours is difficult. Many of them need support to attend meetings; getting time off work to attend meetings during the working week is difficult.”* (R08)
Language	*“I think, for one, it’s just the language, the communication. We’re all used to talking in a scientific lingo or whatever, and, you know, we have to be able to make sure we are all talking the same language so we can understand each other.”* (R07)	*“It also kind of makes things a little bit more challenging because […] especially about all the language that we’re speaking, and I’m not talking about French or English.”* (R04)
Risk of tokenism	*“It needs to be really well figured out how patients would be able to contribute to the grant writing.”* (R10)	*“It’s hard to naturally see how that’s going to work, having a patient there. I mean, it ends up looking very token rather than very practical.”* (R01)
Patient selection	*“Figuring out what do we mean by patient engagement and which patients are we actually including in this process.* […] *getting kind of the right mix of appropriate representation can be certainly challenging.”* (R08) *“The problem is when there is an additional party who will have the patients to bring their agenda.* [sic]” (R05)	*“[There] are all groups that may not be able to volunteer in the same way, then, as a result, you’ll be selecting from voices that have a particular perspective that not totally representative of all patients.* [sic]” (R01)
Power imbalance	*“[T]here is obviously a power difference, power distribution issues, especially if you involve patients; they are vulnerable and, of course, physicians have the relevant power in the health care, so that’s somewhat unequal, so that has to be somehow balanced out.* [sic]” (R06)	*“My concerns are related to transplant research in general, or medical/biomedical research in general, because, well, anyways, it’s a complex issue and I’m not sure if the whole field is as open to the idea. [...] There might be barriers from our part, from the transplant community’s part and the medical community’s part, and that is, you know, I think it is very complex, because this is very deeply related to how we see problems, how, what we see problems, what are the hierarchy in research. What are our own interest and how, through patient engagement, may interfere with those or maybe interact with those.* [sic]” (R03)

PE in research is sometimes seen as time- and energy-consuming, and as an added hurdle in developing a research project*. “[The] PE component of our research adds time, money, energy, potentially even headache[s] and aggravation.”* (R09) Researchers also feared that the research community could be reluctant to engage with patients in research because of the paucity of data on its impacts relative to the effort invested.

The language of researchers was also identified as a challenge to PE in research. Respondents feared that patients would not be able to understand the scientific jargon used by researchers, explaining that they *“often use very arcane language, jargon, and that […] is a huge challenge.”* (R02) However, researchers mentioned that this barrier could be overcome by providing some training for patients.

Researchers acknowledged the risk of tokenism, i.e. adding a PE component to a project solely to fulfil funding agency guidelines without meaningfully engaging with patients. In the words of one respondent, *“You have to be careful not to use patients [...], that’s the risk. I think you have to be extremely vigilant about that, because it’s a fine line, I think, between honestly involving patients, having them truly participating […]. I believe you have to really be vigilant and attentive.”* (R02) Researchers suggested that this could be overcome with a better understanding of PE in research and with training for researchers, patients and grant reviewers. *“I think that’s problematic because there aren’t clear guidelines as to what PE should be, and if you don’t have a clear idea what that means it may mean different things to different people, including different people who are on a grant review panel.”* (R03).

Researchers questioned the process for selecting patients and how the chosen patients could be representative of the patient population. The issue of potential conflicts of interest in reference to patients who might have their own agenda was often mentioned. For example*: “I mean, of course, they’re coming to the table because you want to know, you want the benefit of their experience, but if people have an agenda going into those conversations […] it can become all about their agenda rather than about the actual research program that is being developed, right? So that’s definitely something that needs to be avoided, because that can be very destructive and frankly time-consuming, again, when it comes to the scientist.”* (R04).

Finally, researchers were concerned about a power imbalance due to an unequal relationship between patients and researchers. Patients may feel intimidated by the researchers’ status and because of their own lack of knowledge of research.

#### Researchers’ needs

Lastly, when asked about what should be prioritized regarding PE in the CNTRP, researchers stated that the most important factor should be developing a framework and setting clear objectives. All researchers expressed a clear need for guidance, guidelines and training in order to facilitate the operationalization of this endeavour*. “I think that it needs to be […] focused[,] we need to have an idea of what specifically we’re hoping to achieve through PE and have […] a plan as to what stage in the research process it might be appropriate to bring patients in, and really have a sense of what we’re hoping to achieve with that level of engagement.”* (R08) “*For a researcher, I think probably the most important thing is […] to have some training in the arena of patient engagement in research, and then understanding what that means, and what level of it can be engaged in, and what those engagement activities should be, and how one sort of, for example, builds it into a study protocol.”* (R09).

### Perspectives after the workshop

#### Researchers’ experience and the impact of the workshop

All researchers interviewed after the priority-setting workshop reported that the experience was positive. They emphasized the importance of activities that bring together the various stakeholders in organ donation and transplantation. They enjoyed the opportunity to listen to patients and family members who have personal experience with transplantation and organ donation. Basic science researchers were moved when interacting with patients with whom they did not have contact in their regular activities: “*I thought it was really good and very interesting. It was really nice to engage with patients; being [a] basic scientist, I’m very far away from patients, so usually I don’t see patients, I don’t interact with them. It was really nice to hear their stories and to hear what they thought was important. And I think it definitely opened my eyes as well from a basic science point of view and in […] how can we further engage patients in what we’re doing and [have] their opinions, but also […] inform them better about what we’re doing and how much time things cost and, you know, that it’s a better understanding from both sides.”* (R04).

Researchers found it was very important to hear patients’ perspectives on transplantation research, and the workshop was a tangible way to do that. They noted that patients and health professionals/researchers had different perspectives on the same issues even when the priorities were the same. Here is an example: *“I was sitting there I was thinking wow it’s important to get their views because we are thinking about things in a different way.”* (R06) Meeting with patients adds a personal perspective to the goals of their research, giving them an idea of the impact that their research could have on people’s lives and on families.

Even though researchers were supportive of PE in research before the event, they felt the workshop *“opened their eyes”* to the importance and feasibility of involving patients and gave them ideas on how to do so. *“I definitely realize that it’s possible. […] I knew it was valuable but I was really worried about how this would work. Right? In reality, how [it] was logistically possible, and I can see that now.”* (R07).

Following the workshop, all interviewed researchers planned to engage patients in their research activities, especially as advisors and co-researchers (for grant-writing and protocol design). However, they reiterated the need for guidance, guidelines, training and support in order to facilitate PE in current research programs.

#### Barriers to and facilitators of PE

After the workshop, researchers were concerned with the same potential barriers as those elicited during the pre-workshop interviews. However, researchers’ comments were more patient-focused and more related to challenges faced during the workshop. For example, some researchers worried that patients would not feel confident or entitled to express their views in front of the researcher, who is an expert in the field. One researcher, to support his view, reported an example of this power dynamic during the national workshop: “*I think, on the one hand, […] the academic people—so the clinicians and the scientists by virtue of […] what we do and who we are—there might have been a bit of a power dynamic in the room. And […] for myself, I would certainly feel very free to speak, whereas […] the patient participant might feel more intimidated to speak.”* (R01) Other researchers were concerned about the logistical challenges of partnership with patients: *“[Having] meetings in the evening hours would be difficult, because it’s difficult already across six time zones to get everybody on the same call. So I think that there [are] some, you know, practical things that are barriers to patient participation.”* (R08) Table 2 summarizes the barriers with discussion excerpts.

When asked about potential facilitators, they identified training, allocation of additional resources and institutional support. Given the absence of guidelines on PE, researchers mentioned that training on PE for researchers and participants would facilitate the implementation of PE within the CNTRP. *“I think, one of the things that probably was less clear at the end of the workshop was exactly how to build patient’s participation into the actual research program. Having some better training in the preparation for participants, I think is actually really important to do, so that they feel both comfortable to speak up and also well informed about what it is they are discussing.”* (R01) It also appeared important for participants to provide training to patients on the research and funding processes as well as on abbreviations widely used by researchers. “*Well, there’s always the technical barriers.[...]And then to understand the lingo, we use, unfortunately acronyms you know I think become second nature to us but to have some sort of acronym list and process list of, you know, what a grant is and [what the] CIHR is?”* (R05) Finally, participants also wanted to receive training about how to successfully and meaningfully engage patients in research process.

PE requires time and commitment from all stakeholders, and researchers were aware that resources like time and money should be allocated to these purposes. In the words of one researcher: *“to facilitate time, I think these people need to be paid for their time. […] But figuring out a way of, first of all, a standard amount […] that everyone gets paid and some way of doing this easily… Because most of us don’t have extra research dollars for this. To figure [out] a way to facilitate their hours, travel for face to face.”* (R07) In addition to the need for additional resources, researchers recognized the importance of strategies to promote patients’ voice with the support of the CNTRP*. “I think that the CNTRP need[s] to support the patients as far as travel to meetings and engagement and [all] that [go].”* (R08) The creation of a patient committee in the CNTRP (as suggested in workshop discussions) was seen as a way for the patients to feel engaged and united, and as a place where they could share their experiences. The committee could also help identify potential patient partners in research and offer them training for this role. Table 3 summarizes the potential facilitators with discussion excerpts.

**Table 3:** Potential facilitators of PE in research

Theme	Discussion excerpts
Training	*“Having some better training in the preparation for participants, I think, is actually really important to do, so that they feel both comfortable to speak up and also well informed about what it is they are discussing.”* (R01) *“So some sort of course of training that would bring people up to speed—and it’s not really for me because we can do that too, but I sense that it added some anxiety to them to be in the unknown or uncomfortable with the process—and to have some sort of way of training them so that they’re a little bit more comfortable starting up.*”(R04) *“[Patient training] just so that they start to become experts in, you know, the nuts and bolts of how research is done, which I think will help inform them and us as to how to better design protocols and better involve them in all aspect of research.*”(R08)
Additional resources	*“Need to support the patients as far as travel to meetings and engagement and that.”* (R08) *“Make sure that there’s a resources available online for example, on their website specifically or towards researchers that want to make that patient involvement more active or more a reality in their own research.* *Incorporate patients in the CNTRP model, is to do it in a committee structure that allows patients to be engage together that way whenever we propose ideas or ask them for their input.* [sic]” (R10)
Institutional support	*“Have a patient committee with the patients managing the committee and giving their opinions and all that, and researchers acting as observers.”* (R02) *“[PE] could be integrated within the CNTRP, I think that would be a really good start. […]So, I think one of the ideas that our group had was to sort of create almost like another core, in addition to core 1, 2 and 3, made up of patients; and then you could sort of have these solid group of patient that could be asked if they want to participate in different aspects of the research, but then also it would be sort of its own body within the CNTRP then that would play a role at the annual meetings, that could play a role in the evaluation process and things of that nature.* [sic]”(R09) *“Interviewer: Could the CNTRP actually offer you something to help you out in how to do it [PER]?* *Participant: Yeah, certainly, I think the CNTRP, given its effort, could certainly a sort of vehicle or resource for that for sure. And I think it would be worthwhile making that sort of one of the pillars or strength in CNTRP is patient engagement and patient-relevant research, should make sure that there’s a resources available online for example, on their website specifically or towards researchers that want to make that patient involvement more active or more a reality in their own research.*[sic]*”(R10)*

## Discussion

This qualitative study suggests that CNTRP researchers who participated in this study and in a priority-setting national workshop supported PE in research. Nine out of 10 participants believed that patients should be involved in every step of the research process. This result differs from the results of our national survey, in which researchers (*n* = 88) were asked to choose all aspects of research where patients could have a role to play. Research priority-setting (67.1%), transferring knowledge (65.9%) and evaluating the impact of the research (60.2%) were the steps of research in which researchers believed patients and the public should be involved (unpublished data).

The participants’ views are probably not representative of all CNTRP researchers. Indeed, the fact that they agreed to participate in a priority-setting workshop with patients and caregivers demonstrates their inclination toward PE. Researchers reluctant about PE would probably have declined an invitation to participate in the workshop. In the future, it will be important to collect perspectives of a larger number of CNTRP researchers to include a certain proportion that may be unsupportive of PE, in order to document what keeps researchers from engaging with patients. That being said, these data indicate that there is fertile ground within the CNTRP to engage patients in various areas and steps of the research process. Although some researchers were concerned about PE in basic science research, they acknowledged that despite PE’s added value being less apparent than in the clinical research domain, it could probably serve a purpose.

The national priority-setting workshop strengthened researchers’ views on PE in research and increased their willingness to become involved in PE activities. That being said, they mentioned several barriers to PE in research: lack of funding and time, scientific jargon, patient selection, absence of guidelines and training, and the risk of tokenism. These barriers are similar to what is found in the literature. Brett et al., in a systematic review of 35 studies on the impact of patient involvement on research, showed that lack of time and funding were seen as challenging by researchers. Tokenistic attitudes from researchers, a lack of pre-defined research roles for patients and researchers, and concerns about patients’ hidden agendas were reported as challenges and barriers to genuine PE in research. [17] Another systematic review conducted by Domecq et al. showed that the extra time and funding needed to conduct research with patient partners and the risk of tokenism were reported as barriers to PE. Scope creep, or the concern that engaging patients will bring irrelevant issues into the research program, was also cited as a barrier. [18] Scope creep was not a concern mentioned by the participants of this study. Perhaps that could be explained by a social desirability bias given that our research team is also leading the patient partnership strategy. [15]

As mentioned earlier, tokenism was frequently cited by researchers as a concern with PE. In 2016, Hahn et al. published the results of a workshop with researchers, patients and clinicians to discuss ways of moving beyond tokenism in PE. They identified three domains in the development of genuine engagement with patients: (i) engagement methods and structure (e.g. adequate stakeholder diversity, power sharing and co-leadership, scheduling allowing enough time to accomplish tasks, education and training, defining of roles, clear expectations, use of appropriate language); (ii) intent (e.g. involving patients in determining objectives, reciprocal learning); and (iii) relationship-building (e.g. building mutual trust and respect, promoting and nurturing partnerships before, during and after projects). [19] Moreover, Kirwan et al. recently published the principles that should guide PE in research to prevent tokenism, namely: (i) implementing supportive institutional policies; (ii) fostering and encouraging positive attitudes toward partnership among researchers and patients; (iii) establishing a culture of trust, respect, reciprocity and co-learning; (iv) offering adequate researcher and patient training; (v) taking into account resources needed to establish a successful partnership; and (vi) valuing partnership through all stages of research. [20] Some of these principles were explicitly mentioned by participants. The participants in this study identified institutional support, training of researchers and patients, and adequate resources as facilitators to PE in research. That being said, the above principles will need to be considered in the development and implementation of a PE strategy within the CNTRP.

Training for researchers and patients was frequently listed as a facilitator of PE. Participants mentioned that patients should receive training about the basic language of research, and about research and funding processes. Researchers wanted to get more training on how to successfully and meaningfully engage patients in research. The content of said training, however, was not clearly defined by participants. For patients, should this training be about technical aspects of research? According to Abma and colleagues, a purely technical training for patients could give them the impression that they would be listened to and taken seriously if they spoke the same language as the researchers. [21] For researchers, should there be training, or rather mechanisms, to improve communication and collaboration skills? The CNTRP has co-developed training materials for patients and researchers (webinars and presentations for face-to-face meetings) addressing the basics of patient engagement (e.g. types of engagement, principles, winning conditions, challenges) and of the research and funding processes. These materials are currently being evaluated through our assessment study of patient engagement within the CNTRP and will help to inform the future development of training.

Lastly, the researchers did not mention any specific impact of PE that should be measured. Brett and colleagues have conducted systematic reviews of the impact of patient involvement in research in health and social care. [17, 22] They reported positive impacts for patients and researchers (empowerment, increasing the relevance of the research question, facilitating recruitment, etc.) that are similar to the benefits and anticipated impacts mentioned by the researchers who participated in this study. However, there was no mention of how to measure the impact of PE in research. According to Staley, measuring the impact of PE in research is particularly challenging, since this impact is unpredictable within any research project. Also, it is impossible to determine a quantitative tool to measure the impact given the different forms that PE in research can take. [23] Indeed, involving a patient in the research design will have a different impact than involving them in knowledge dissemination and transfer. The CNTRP is currently assessing the patients’ and researchers’ experiences of patient partnership in research. This assessment will allow us to better understand when, where, why and how patient partnership is valuable and relevant in transplantation and organ donation research. The empirical data will contribute to the improvement of existing partnerships and will help us discuss the perceived impacts of patients and research teams partnering together. Rigorous evaluation would not only help the CNTRP communicate the relevance and effectiveness of patient partnership, but enhance patients’ and researchers’ ability to strengthen their working relationships and implement effective collaborative projects.

## Conclusion

The CNTRP researchers who participated in the national workshop on priority-setting had very positive attitudes toward PE in research. Patients’ experiential knowledge was viewed as a way to enhance the relevance and quality of medical research within the CNTRP. According to the interviews, patients should be involved in every step of the research process, and although some considered clinical research as most suited for PE, others acknowledged that patients could even play a role in biomedical research. Lack of funding and time, scientific jargon, tokenism and patient selection are viewed as challenges of and potential barriers to PE. On the other hand, training, resources and institutional support were perceived as facilitating factors.

Given this fertile ground for PE in research, in 2016 the CNTRP created the Patient Researcher Partnership Platform, or Core 4. Core 4 is co-led by a patient and researcher. [10] This platform oversees the integration of patients as co-leads in three CNTRP projects (including a biomedical project) and as patient researchers in three studies. The mandate of Core 4 is to: give the patient a voice and be a catalyst for cultural transformation; align CNTRP priorities and activities to ensure that the CNTRP remains relevant to the needs of patients; contribute proactively to the design and implementation of the patient-researcher partnership strategy; and assist and support research and project teams in integrating patients. In the next year, we will assess this partnership and document its impact within the research network of the CNTRP.

## Abbreviations

CNTRP: Canadian National Transplant Research Program
PE: Patient engagement
